# Influence of the coiling porosity on the risk reduction of the cerebral aneurysm rupture: computational study

**DOI:** 10.1038/s41598-022-23745-1

**Published:** 2022-11-09

**Authors:** Armin Sheidani, M. Barzegar Gerdroodbary, Amin Poozesh, Amir Sabernaeemi, Sajad Salavatidezfouli, Arash Hajisharifi

**Affiliations:** 1 Mechanical Engineering Department, Politecnico di Milano, Milan, Italy; 2 Department of Mechanical Engineering, Babol Noshirvani University of Technology, Babol, Iran; 3 Department of Aerospace Engineering, K.N. Toosi University of Technology, Tehran, Iran; 4 Department of Space, Earth and Environment, Chalmers University of Technology, Gothenburg, Sweden; 5 Mathematics Area, MathLab, International School for Advanced Studies (SISSA), Trieste, Italy

**Keywords:** Biomedical engineering, Mechanical engineering

## Abstract

The formation and progress of cerebral aneurysm is highly associated with hemodynamic factors and blood flow feature. In this study, comprehensive efforts are done to investigate the blood hemodynamic effects on the creation and growth of the Internal Carotid Artery. The computational fluid dynamic method is used for the visualization of the bloodstream inside the aneurysm. Transitional, non-Newtonian and incompressible conditions are considered for solving the Navier–Stokes equation to achieve the high-risk region on the aneurysm wall. OSI and WSS of the aneurysm wall are compared within different blood flow stages. The effects of blood viscosity and coiling treatment on these factors are presented in this work. Our study shows that in male patients (HCT = 0.45), changing the porosity of coiling from 0.89 with 0.79 would decreases maximum OSI up to 75% (in maximum acceleration). However, this effect is limited to about 45% for female patients (HCT = 0.35).

## Introduction

The major cause of premature and abnormalities deaths throughout the world is Cardiovascular diseases (CVDs). World Health Organization (WHO) reported that annual death related to CVDS is about 18 million peoples and this disease is known as the main threat for human life in the future. Among the different CVDs, a Cerebral Aneurysm (CA) is a conventional disease which causes severe unwanted effect on human life^1,2^. A Cerebral Aneurysm (CA) is a thin or weak sac on an artery in the brain that bulges or balloons out and fills with blood and 3–5% of peoples has this disorder. An intracranial or cerebral aneurysm is an unusual main enlargement of an artery in the brain and it is mainly initiated when the internal muscular layer of a blood vessel wall becomes weak^3,4^. The rupture of this arterial wall threating the human life by coma, stroke or death. Although significant advances have been achieved in surgical techniques, high rates of death were reported by aneurysm rupture in recent years.

The endovascular coiling and microsurgical clipping are current treatment selections and both of them have side effects and high risk^5–7^. Available resource show that the size of most CA is small and about 70% of these CA did not rupture. Researchers have reported that the aneurysmal dilatation at the apex and local weakness are the main reasons for the formation of the CA. A finite element application of arterial wall evolution and remodelling with application to abdominal aortic aneurysms were done to investigate the risk of this disorder^8–10^. Because of the impingement of main blood stream, hemodynamic force applied on the artery and this is significant factor for degradation of the flexible membrane. Although Intracranial aneurysms have been recognized as a system of rare family, they are not known as sporicidal wounds.

Three main treatments for this disease are clipping, coiling and stenting. Due to surgical issue, the first treatment is not always used. In fact, clipping is widely replaced by the coiling technique. Besides, the selection of the treatment technique is mainly done over the analysis of the geometrical aspects of aneurysm^11,12^. Meanwhile, aneurysm conditions and locations are also considered for the evaluation of the treatment. In Guglielmi Detachable Coil (GDC) procedure, endovascular coiling is done by an interventional neuro-radiologist with high skill in interventional radiology. Although the ratio of accomplishment in clipping technique is higher than in coiling, some difficulties, such as, haemorrhage, bleeding, high risk, and incomplete occlusion coiling does not happen by application of coiling. In fact, surgeons could manage the coil inside the aneurysms^13,14^. After inserting the coil inside the aneurysm, light electricity is streamed in the coil. Although this technique seems efficient for aneurysm treatments, there are still some disadvantageous and it is not recommended for the aneurysm with wide neck.

Due to importance of the aneurysm feature, hemodynamic analysis of the blood stream within the cerebral aneurysm becomes important. In fact, the diagnosis of the high risk section on the wall of the aneurysm is highly significant for the coiling process. It should be considered that the blood stream inside the arteries follows the rhythm of heart pulse and this has high influence on the growth and rupture of the aneurysms. Although several works have been developed for the hemodynamic study of the blood stream inside the aneurysms^15–17^, the most previous works just select superficial aneurysm model for case studies. Meanwhile, correct characteristics of blood stream is not also considered for their evaluations.

**Figure 1:**
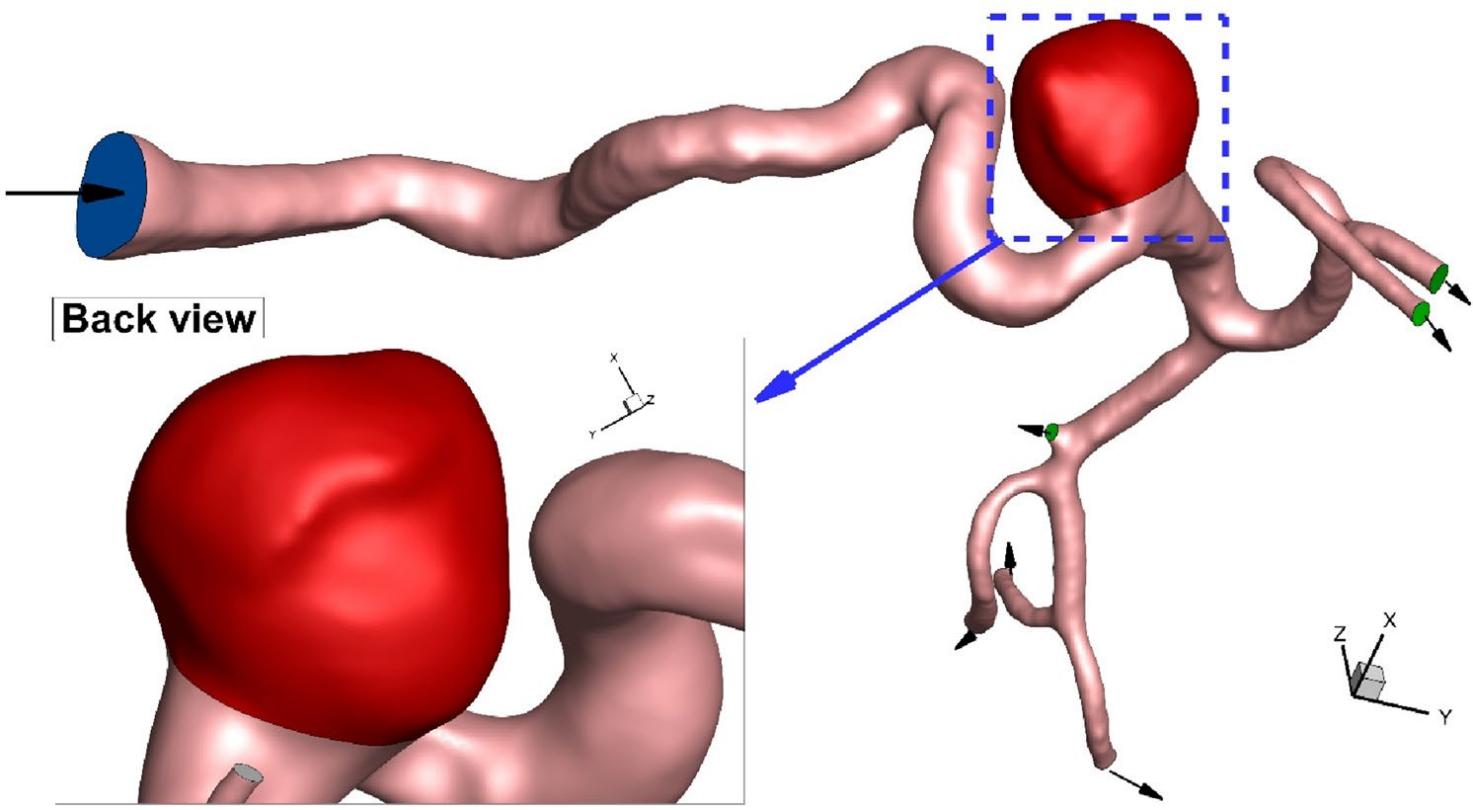
ICA aneurysm.

In current work, hemodynamic features of the blood stream inside the real cerebral aneurysm have been investigated to diagnose the main factors for the progress of the aneurysm and/or its rupture (Fig. 1). Computational fluid dynamic (CFD) is used for simulation of the blood flow. The effective of pulse blood flow is also considered in this work by transient study of the blood stream. The effects of the blood viscosity and coiling percentage inside the aneurysm are also investigated. Wall shear stress and OSI are also compared in different blood flow rate to find the high-risk region for rupture.

## Applied technique for modelling

The computational study of the blood stream inside the artery is done via CFD method. Navier–Stokes equations are solved while it assumed that blood flow is transient, incompressible and non-Newtonian^18^. Casson model is used for the estimation of blood viscosity and stress on aneurysm wall with haematocrit value of 40%. The selected aneurysm (internal carotid artery) geometry is obtained from Emory University and details are available in Ref.^19^. Since the blood stream velocity is not high, simple algorithm is used for the modelling of blood stream^20–23^.

**Figure 2:**
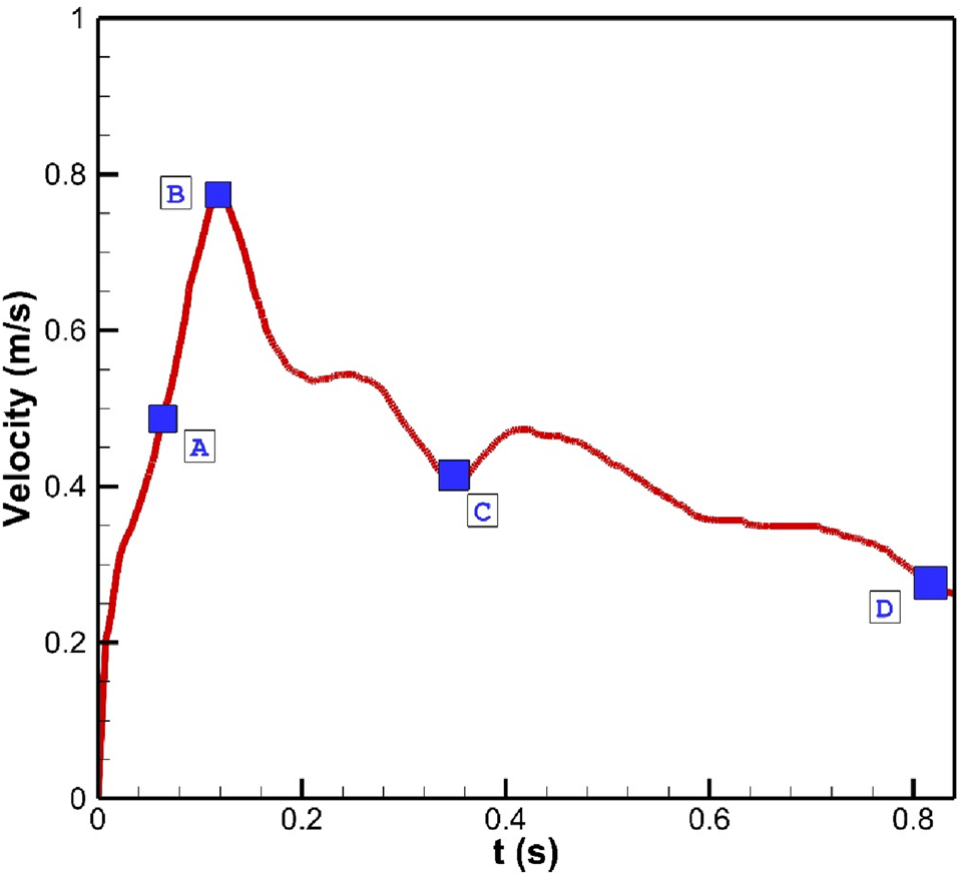
Transient blood flow.

In this work, the blood stream flow profile for inlet is obtained from profile introduced by Boccadifuoco^24^ (Fig. 2). As displayed in this figure, four distinct points are selection for transient effects of blood pulsatile flow. As shown in this figure, the maximum acceleration (A), peak systole (B), maximum deceleration (C), early diastole (D). The coiling of the model is employed via the porosity inside the aneurysm and the details of selected porosity is obtained from Mitsos et al.^25^. In this work three main factors of Wall Shear stress (WSS), pressure and OSI are compared in different temporal and operational conditions. OSI is calculated, via following equation:

1 $$WSS=\mu {\left(\frac{\partial u}{\partial y}\right)}_{y=0},$$

2 $$OSI=\frac{1}{2} \left(1-\frac{\left|{\int }_{0}^{T}{WSS}_{i} dt\right|}{{\int }_{0}^{T}\left|{WSS}_{i}\right| dt}\right),$$

where *u* is tangential velocity, *t* is time, and *T* is the duration of the cycle.

**Figure 3:**
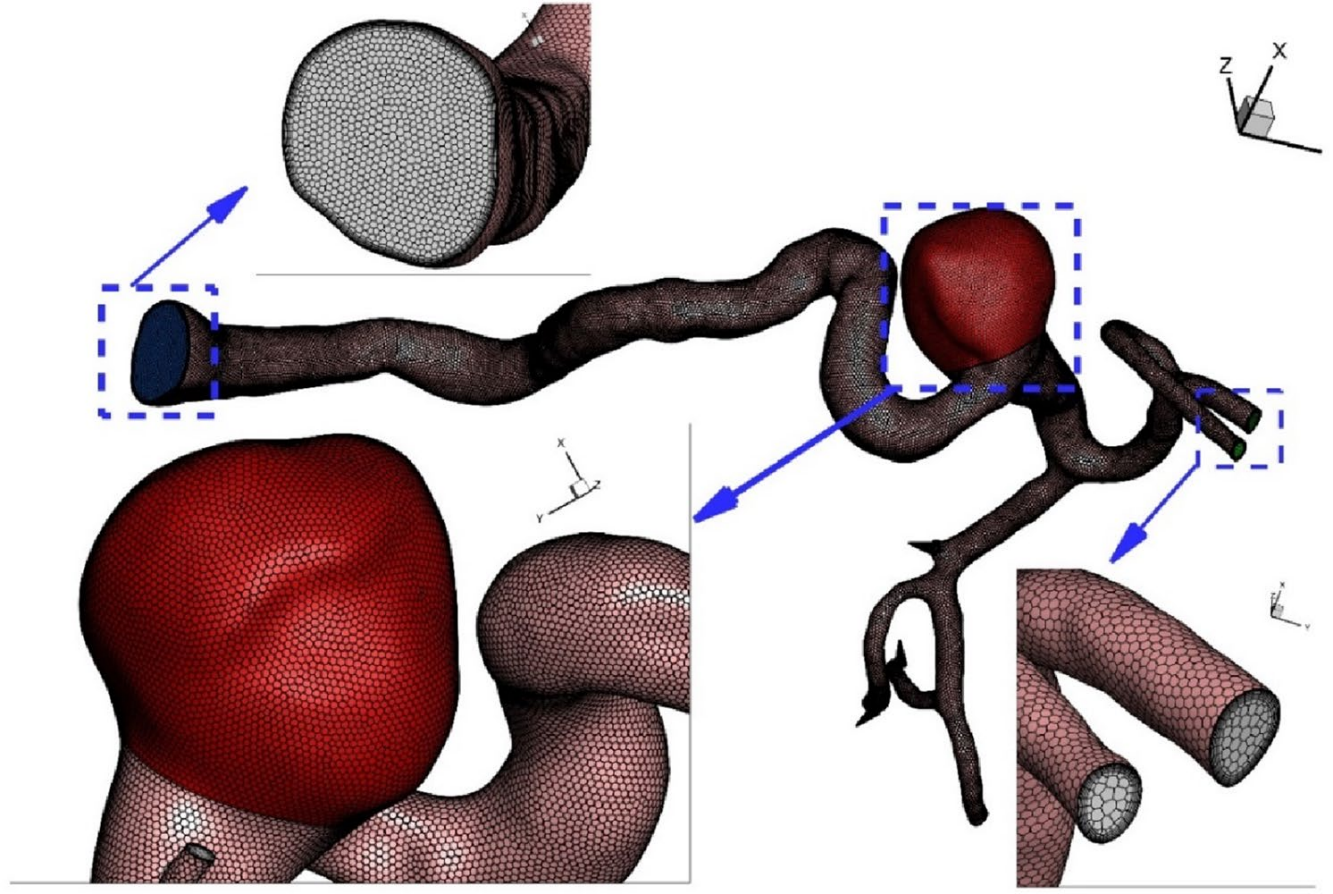
Grid production.

Computer simulations have been widely used for the simulation of biomechanical devices^26–32^. Due inaccessibility, this technique is the best option for analysis of the blood hemodynamic^33–40^. This The grid generation is also required for the control volume approach^41–49^. As depicted in Fig. 3, unstructured grid is produced for the selected ICA. For grid independency, we also examined four grids sizes for specific condition and Table 1 demonstrates the impacts of different grids on the velocity magnitude at neck of aneurysm. Finally, fine grid with 1,624,000 cells are chosen for our study.

**Table 1:** Details of used grids.

	Cells	Average blood velocity at inlet of sac (maximum acceleration)	Average blood velocity at inlet of sac (peak systolic)
Coarse	722,000	0.342	0.51
medium	1,120,000	0.361	0.54
fine	1,624,000	0.379	0.56
Very fine	2,064,000	0.382	0.561

## Results and discussion

**Figure 4:**
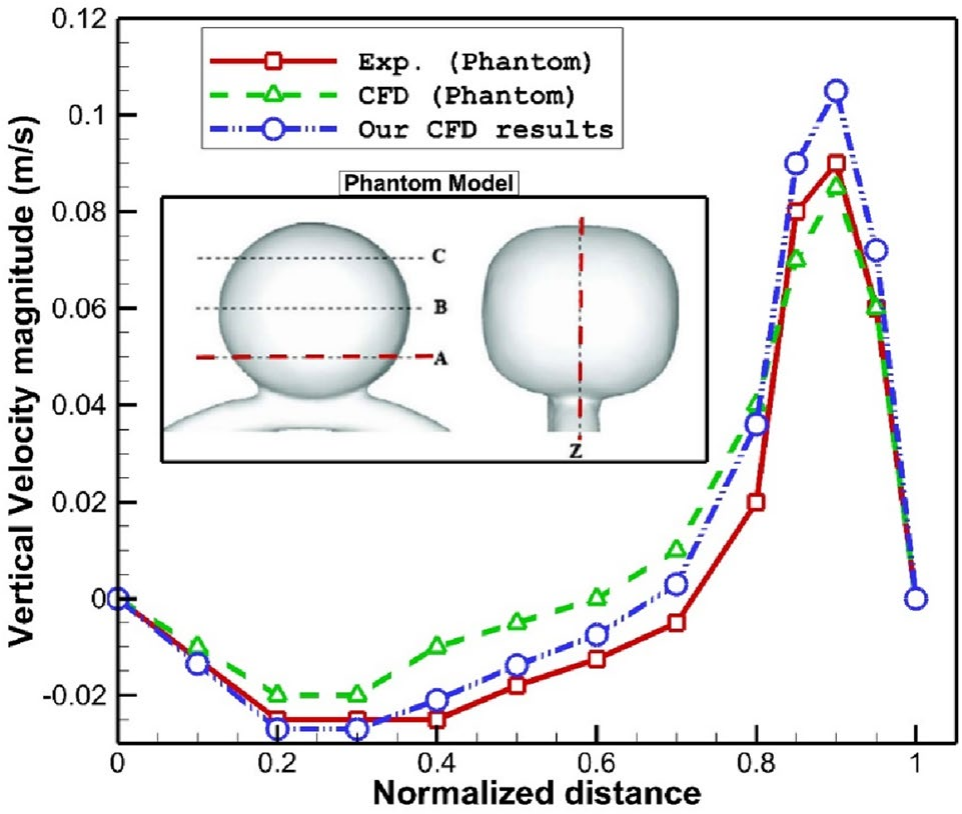
Validation.

To ensure about the results, model developed by Hoi et al.^26^ is selected for the validation of our method is done. As demonstrated in Fig. 4, the phantom model developed by Hoi et al.^26^ is simulated and the vertical velocity on the cross-section of plane A and Z are compared with our data. It is found that velocity trend of our results agree well with that of experimental works.

**Figure 5:**
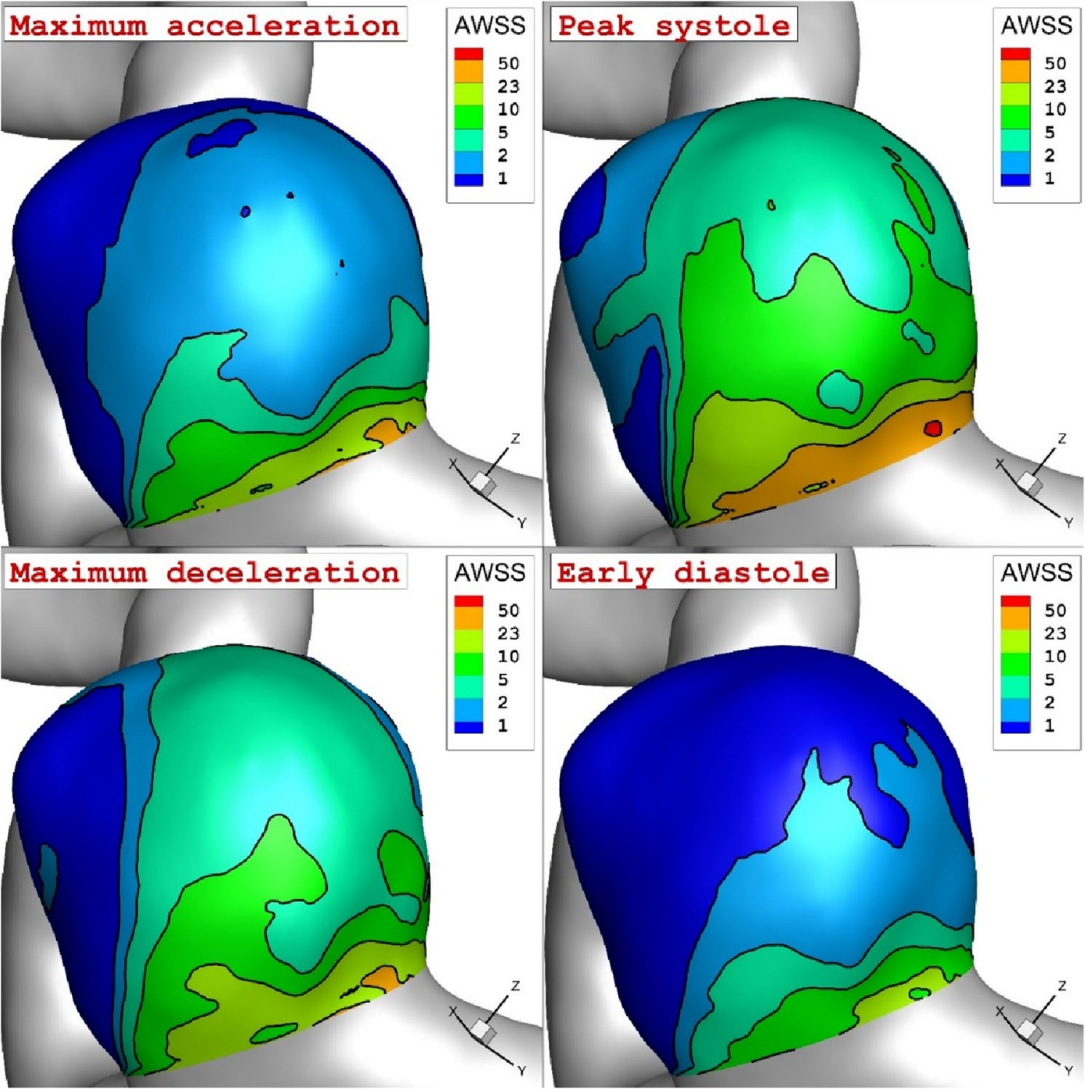
The variation of the WSS on the aneurysm wall in various instants.

The main attention of this work is to demonstrate the impacts of blood flow pulsation pattern on the main factors i.e. wall shear stress, pressure and OSI on aneurysm wall. Figure 5 demonstrates the wall shear stress on aneurysm wall at four stages of the maximum acceleration (A), peak systole (B), maximum deceleration (C), early diastole (D). The comparison of the AWSS on aneurysm wall in different time instants clearly demonstrates the impacts of the blood mass flow rate on the growth and rupture of the cerebral aneurysm. It is found that the maximum AWSS happens at the neck of the aneurysm sac. The change of the WSS also indicates that the region with high curvature has maximum wall shear stress. In the following section, we would present more details about the pattern of the WSS change in our selected model.

**Figure 6:**
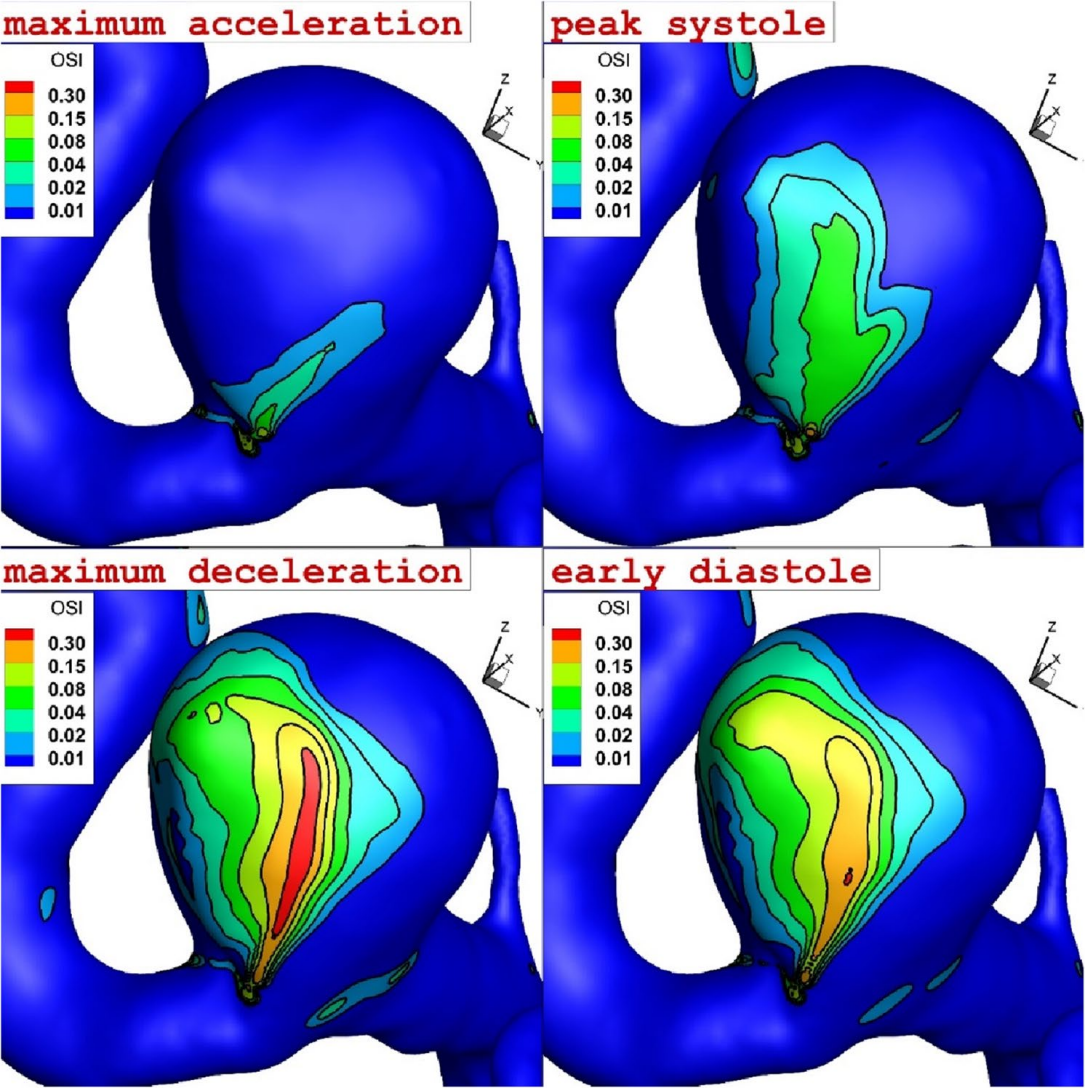
The change of the OSI on the aneurysm wall in four instants of maximum acceleration, peak systolic, maximum deceleration and early diastole.

The OSI value is a reliable factor for the evaluation of the WSS and the detection of high-risk region on the aneurysm wall. Figure 6 demonstrates the growth and change of the OSI in different instants while the blood HCT and coiling porosity are 0.35 and 0.79, respectively. It is noticed that the initiation of high OSI occurs in the neck of aneurysm while it extends into the dome of the aneurysm. The comparison of OSI and WSS contour demonstrates that the maximum OSI occurs in the maximum deceleration while maximum WSS reaches maximum value in peak systolic.

**Figure 7:**
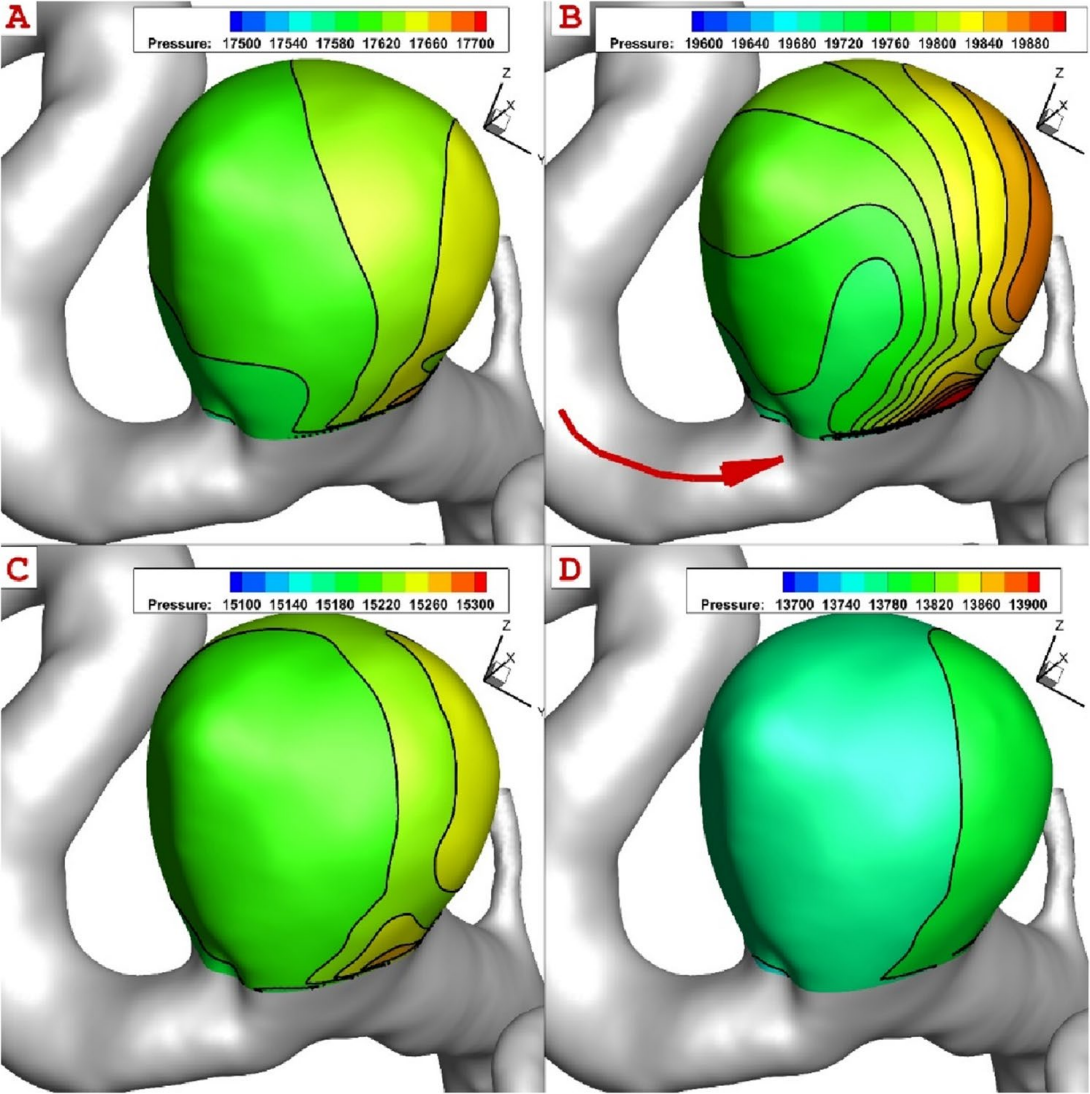
The comparison of the pressure distribution on the aneurysm sac in different instants.

The comparison of the pressure on the aneurysm wall indicates that the angle of blood direction with inlet of sac is highly important for the creation of high-pressure region on sac wall (Fig. 7). The pressure distribution also shows that the incoming blood pressure becomes maximum on the top end of aneurysm sac at peak systolic instant. Besides, the pressure of the blood at neck is high enough to consider this region as high-risk region.

**Figure 8:**
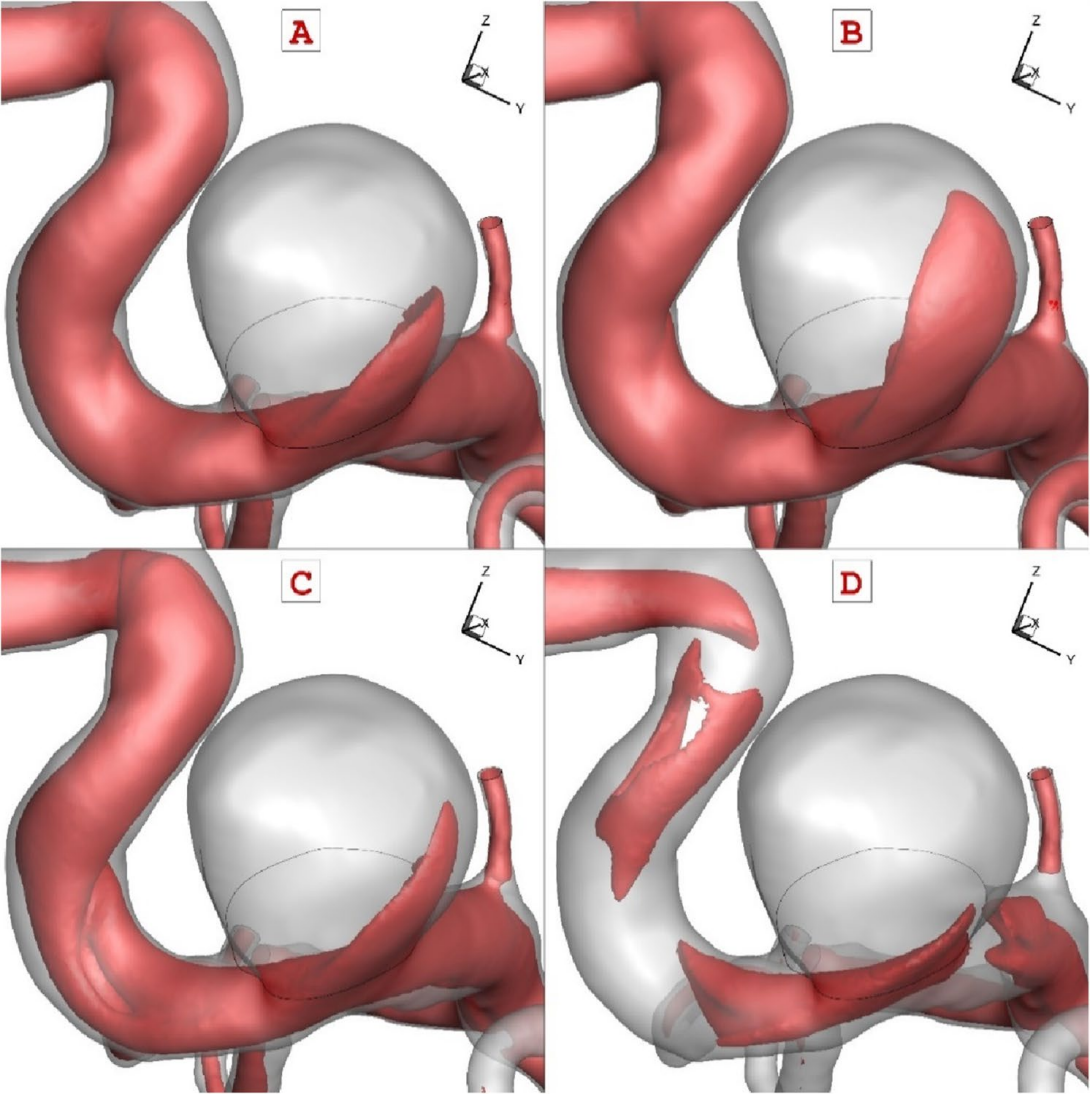
The blood flow feature in various instants (velocity magnitude iso-surface = 0.4 m/s).

The effects of blood flow rate on the hemodynamic of the blood flow is presented in Fig. 8. In this figure, iso-value of blood velocity magnitude (u = 0.4 m/s) is demonstrated to perceive the feature of the blood stream inside the aneurysm sac at different stages in the blood cycle. According to our results, the blood iterance plays main role on the shape of the aneurysm nearby neck.

**Figure 9:**
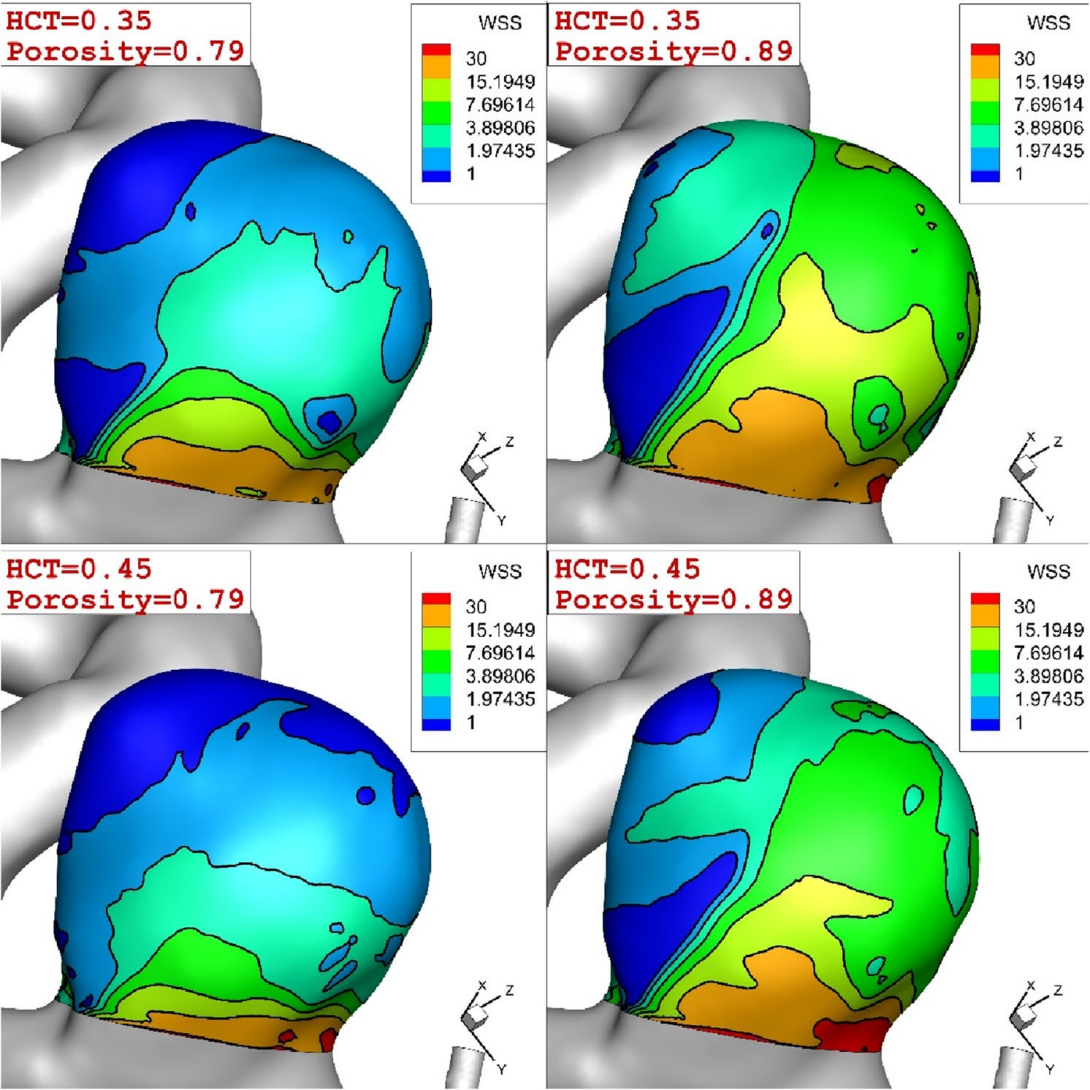
Comparison of the WSS on ICA for different coiling porosities and blood HCTs.

Due to important of the coiling technique and blood viscosity, we compare these factor on the aneurysm wall in peak systolic condition. Figure 9 illustrates the change of WSS on the aneurysm wall for different coiling porosities (0.79 and 0.89) and blood HCTs of 0.35 and 0.45. Distribution of the WSS confirm that the effects of blood stream on the aneurysm wall declines when the porosity of the coiling is increased. Besides, it is found that the effect of blood viscosity is not substantial on the WSS distribution on the aneurysm wall.

**Figure 10:**
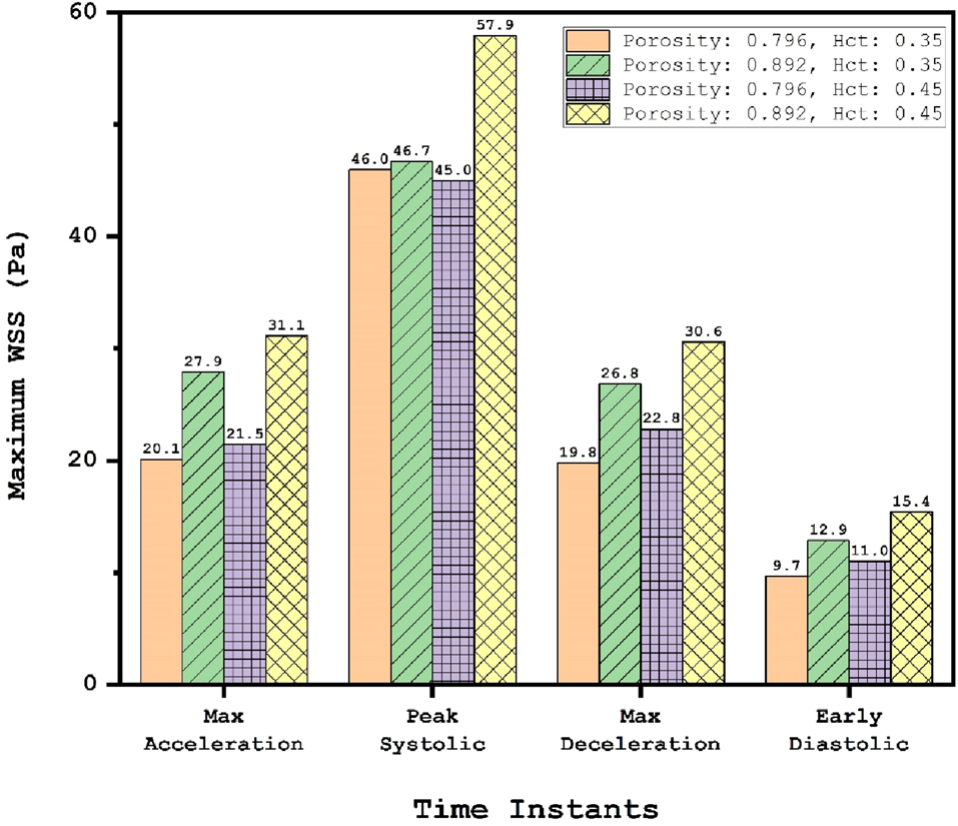
Max WSS (Pa) on Aneurysm in different time instants.

Figure 10 presents quantitative comparison to disclose the effects of coiling and blood haematocrit (HCT) on maximum value of WSS on the aneurysm wall. As expected, maximum value of WSS happens in peak systolic where the effects of coiling are more visible than HCT. In maximum acceleration and deceleration instants, the effects of blood HCT is about 8.5% and found that WSS of female patient (HCT = 0.35) is always lower than that of male (HCT = 0.45).

**Figure 11:**
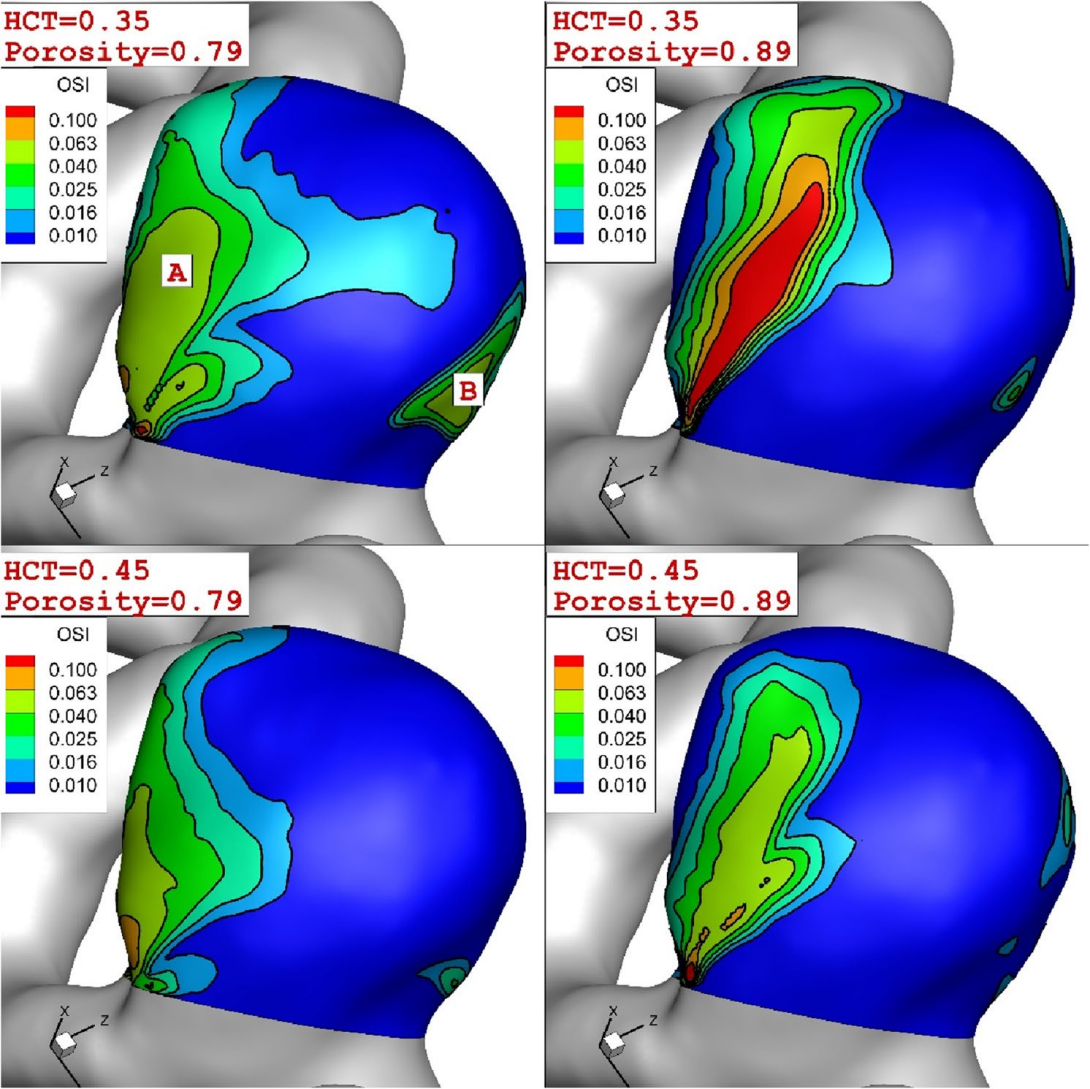
Comparison of the OSI on ICA for different coiling porosities and blood HCTs.

Due to importance of OSI value, this factor is also compared in these conditions for the chosen ICA. Comparison of OSI for these conditions indicates that there are two high-risk regions on the surface of the aneurysm (as demonstrated by A and B in Fig. 11). Results show that the increasing porosity fraction considerably extends zone A while zone B is limited in the same condition. It is observed that increasing the HCT decreases the intensity of the OSI on the wall of the aneurysm.

**Figure 12:**
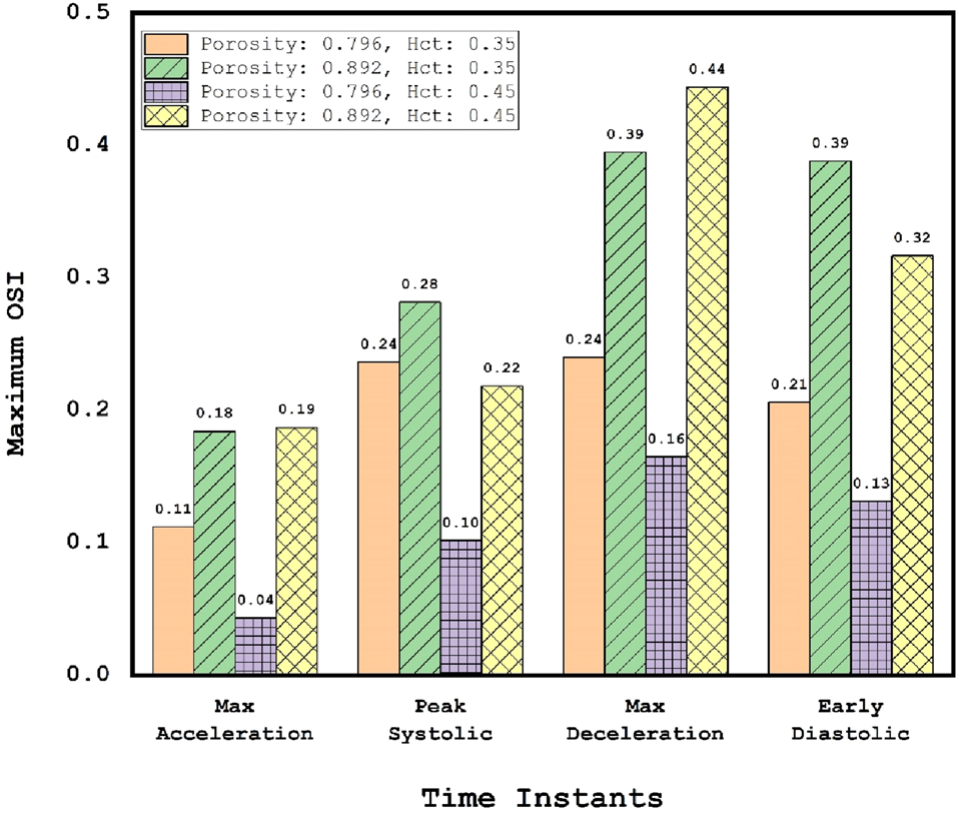
Impacts of coiling porosity and blood HCT on the maximum WSS in different stages.

Quantitative comparison of the maximum OSI also confirm the role of porosity on the reduction of the WSS in different time stages (Fig. 12). In high HCT (HCT = 0.45), changing the porosity of coiling from 0.89 with 0.79 would decline maximum OSI up to 75% (in maximum acceleration). However, this effect is limited to about 45% for the low HCT (HCT = 0.35).

**Figure 13:**
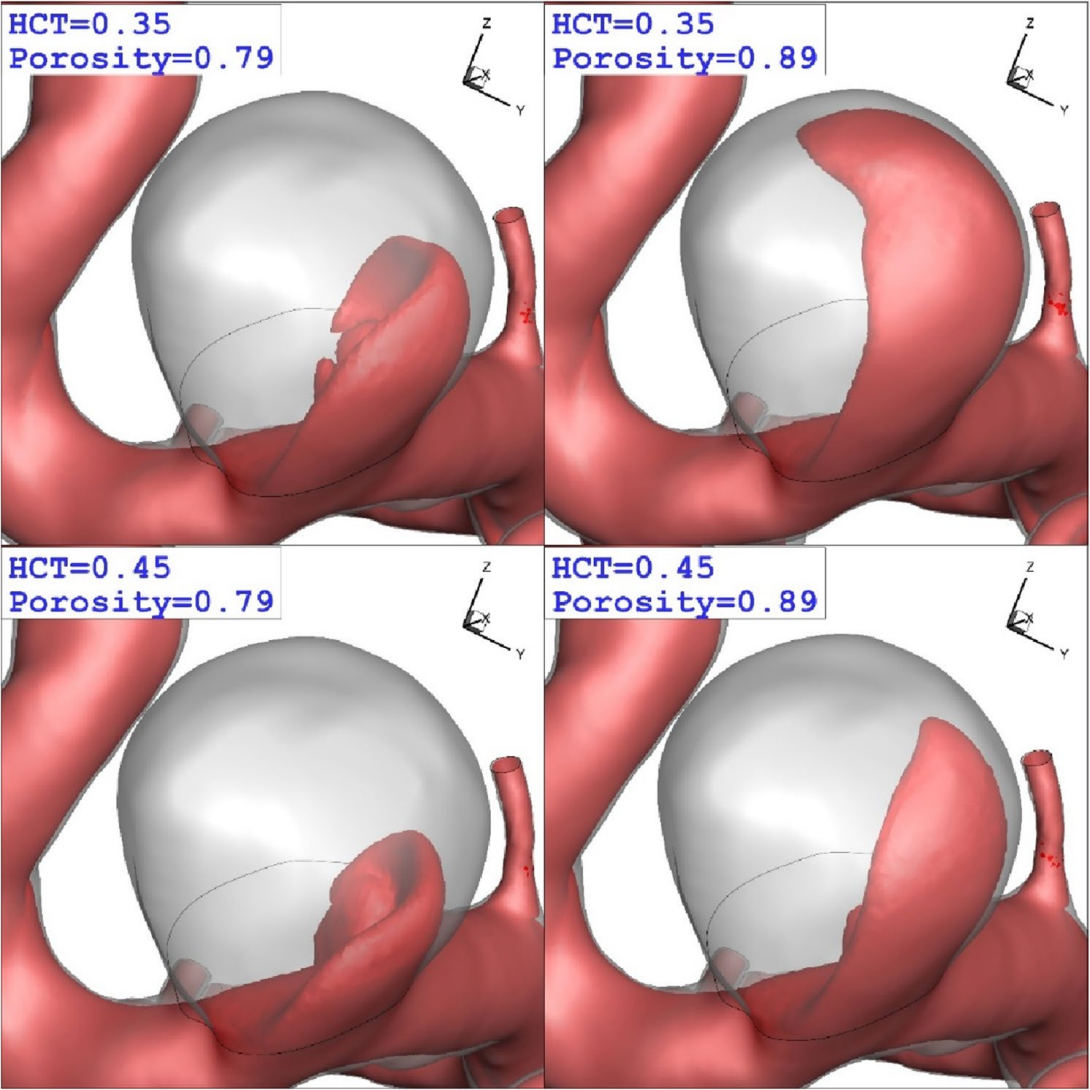
The iso-value of the blood velocity (u = 0.3 m/s).

**Figure 14:**
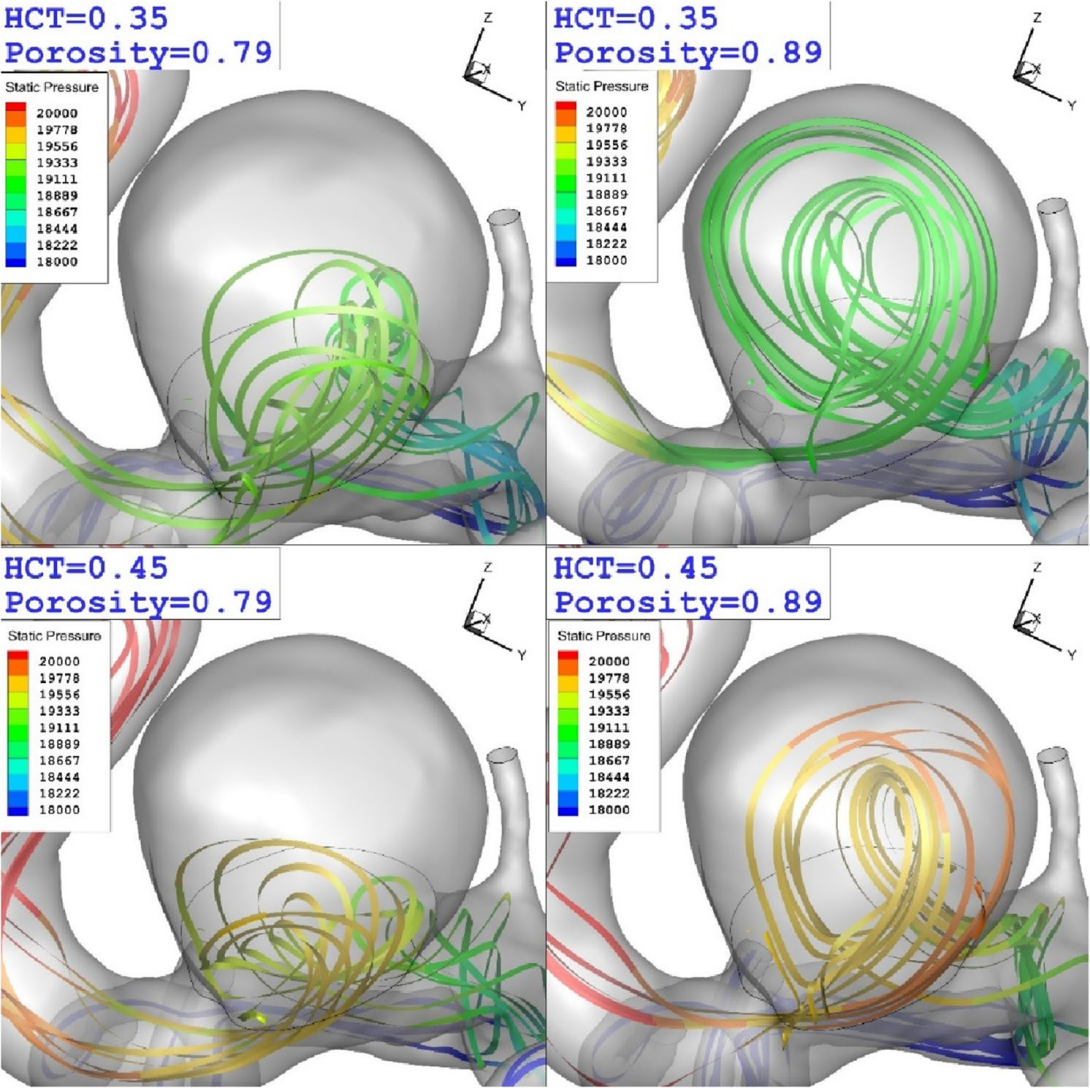
The feature of the blood stream inside aneurysm sac in different coiling porosity and blood HCTs.

The impact of coiling porosity and blood HCT on the blood iso-velocity are depicted in Fig. 13. The comparison of the blood hemodynamic indicate that the lower porosity (equivalent to higher permeability) considerably limited the blood inflow into the aneurysm sac. Besides, it also declares that the blood viscosity is not effective on blood inflow into the aneurysm. Figure 14 depicts the flow stream inside the aneurysm. The comparison of the flow stream shows that the pressure of incoming blood into the aneurysm decreases in male patients (HCT = 0.45).

**Figure 15:**
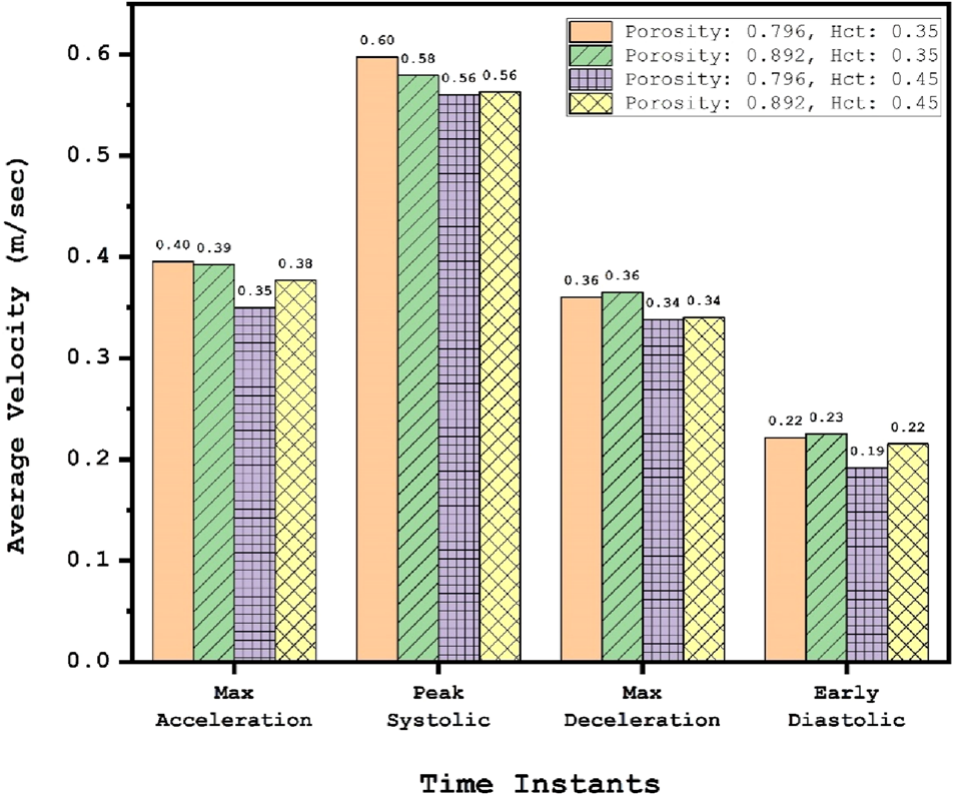
Comparison of the average velocity of the blood flow into the aneurysm sac for different condition and time instants.

Figure 15 illustrates the average velocity entering to the sac of aneurysm in these four conditions. The achieved results indicate that effects of porosity and HCT on the average blood velocity is limited due to geometrical aspects of the selected aneurysm. In fact, the angle of the incoming blood stream with normal of neck face is high and this diminish the impact of aneurysm condition on the velocity of blood inflow.

## Conclusion

This work has tried to evaluate the risk of aneurysm rupture for specific ICA in various temporal conditions. Effects of blood pulsate flow on production and growth of the high risk-region. Analyses of wall shear stress, pressure and OSI are done to disclose the hemodynamic effects on the risk of aneurysm rupture. Besides, the effects of coiling and blood viscosity on these factors are also investigated in various conditions. Blood stream inside the aneurysm and shear stress on the wall of the aneurysm are compared for these blood and aneurysm conditions. Our results indicate that blood HCT has limited impact on the growth and rupture of aneurysm while coiling porosity could decrease OSI and WSS about 50% on the aneurysm wall.

## Data Availability

All data generated or analysed during this study are included in this published article.
